# Supplementary material for: Moderating Effects of BDNF Genetic Variants and Smoking on Cognition in PTSD Veterans
Source: Biomolecules. 2021 Apr 26;11(5):641. doi: 10.3390/biom11050641 (PMC8146493; doi:10.3390/biom11050641)
Supplement: Supplementary file 1 [file biomolecules-11-00641-s001.zip › biomolecules-1156184-supplementary.pdf]

biomolecules

MDPI

Supplementary Table 1. Distribution of BDNF rs6265 and BDNF rs56164415 polymorphisms between smokers and non-smokers in control subjects and veterans with PTSD. The data is shown as total number N and frequency.

	Polymorphism	Genotype / Allele	Smoking		Statistics
			Yes	No	
			N	N	
Control subjects (N=120)	BDNF rs6265	AA	1	3	χ^2=2.035; df=2; p=0.361
		GA	25	22	
		GG	41	28	
		A carriers	26	25	x2=0.847; df=1; p=0.357
		GG	41	28	
		A	27	28	χ^2=1.315; df=1; p=0.251
		G	107	78	
	BDNF rs56164415	CC	55	49	χ^2=3.330; df=2; p=0.189
		CT	2	0	
		TT	10	4	
		T carriers	12	4	χ^2=2.750; df=2; p=0.097
		CC	55	49	
		C	112	98	χ^2=4.258; df=1; **p=0.039**
		T	22	8	
Subjects with PTSD (N=519)	BDNF rs6265	AA	8	4	χ^2=0.738; df=2; p=0.691
		GA	125	57	
		GG	211	114	
		A carriers	133	61	x2=0.718; df=1; p=0.397
		GG	211	114	
		A	141	65	χ^2=0.539; df=1; p=0.463
		G	547	285	
	BDNF rs56164415	CC	250	136	χ^2=1.811; df=2; p=0.404
		CT	91	37	
		TT	3	2	

	T carriers	94	39	χ^2=1.546; df=1; p=0.214
	CC	250	136	
	C	591	309	χ^2=1.144; df=1; p=0.285
	T	97	41	

BDNF - brain derived neurotrophic factor; χ^2 – chi-square test

Supplementary Table 2. PANSS cognitive subscale scores in control subjects and veterans with PTSD, depending on the smoking status and BDNF rs6265 and BDNF rs56164415 polymorphism. The data is shown as median and interquartile range (25th; 75th percentile), while p values in bold represent statistical significance.

Smoking	Polymorphism	Genotype / Allele	PANSS cognitive subscale scores			
			Control subjects	Statistics	Subjects with PTSD	Statistics
Smokers	BDNF rs6265	AA	6 (6; 6)	H=8.373; df=2; **p=0.015**	6 (5; 8)	H=6.647; df=2; **p=0.036**
		GA	4 (4; 4)		7 (6; 9)	
		GG	4 (4; 4)		7 (5; 8)	
		A carriers	4 (4; 4)	U=493.0; p=0.361	7 (6; 8)	U=12090.5; **p=0.029**
		GG	4 (4; 4)		7 (5; 8)	
		A	4 (4; 4)	U=1420.5; p=0.813	7 (5; 8)	U=35181.5; p=0.104
		G	4 (4; 4)		7 (5; 8)	
	BDNF rs56164415	CC	4 (4; 4)	H=0.318; df=2; p=0.853	6 (5; 8)	H=10.729; df=2; **p=0.005**
		CT	4 (4; 4)		8 (6; 8)	
		TT	4 (4; 4)		6 (5; 7)	
		T carriers	4 (4; 4)	U=316.5; p=0.695	8 (6; 8)	U=9327.0; **p=0.003**
		CC	4 (4; 4)		6 (5; 8)	
		C	4 (4; 4)	U=1194.0; p=0.685	7 (5; 8)	U=24113.5; **p=0.011**
		T	4 (4; 4)		8 (6; 8)	
Non smokers	BDNF rs6265	AA	4 (4; 4)	H=2.010; df=2; p=0.366	6 (5; 7)	H=0.869; df=2; p=0.647
		GA	4 (4; 4)		6 (5; 8)	
		GG	4 (4; 4)		6 (5; 8)	
		A carriers	4 (4; 4)	U=320.5; p=0.251	6 (5; 8)	U=3262.0; p=0.495
		GG	4 (4; 4)		6 (5; 8)	
		A	4 (4; 4)	U=1045.0; p=0.462	6 (5; 8)	U=8681.5; p=0.424
		G	4 (4; 4)		6 (5; 8)	
	BDNF rs56164415	CC	4 (4; 4)	U=53.0; **p=0.001**	6 (5; 8)	H=8.418; df=2; **p=0.015**
		CT	.		7 (6; 8)	
		TT	5 (4; 6)		9 (7; 10)	

	T carriers	5 (4; 6)	U=53.0; **p=0.001**	7 (6; 8)	U=1892.5; **p=0.006**
	CC	4 (4; 4)		6 (5; 8)	
	C	4 (4; 4)	U=212.0; **p<0.001**	6 (5; 8)	U=4614.5**; p=0.004**
	T	5 (4; 6)		7 (6; 8)	

BDNF - brain derived neurotrophic factor; H – Kruskal-Wallis test statistics; PANSS - Positive and Negative Syndrome Scale; U – Mann-Whitney test statistics value

Supplementary Table 3. ROCF Copy, Immediate and Delayed Recall Test scores in all veterans and in veterans with PTSD, depending on the smoking status and BDNF rs56164415 polymorphism. Results are shown as median and interquartile range (25th; 75th percentile), while p values in bold represent statistical significance.

Polymorphism	Genotype / Allele	ROCFT Copy	Statistics	ROCFT Immediate Recall	Statistics	ROCFT Delayed Recall	Statistics
All veterans BDNF rs56164415	CC	20 (20; 20)	H=1.036; df=2; p=0.596	13 (11; 15)	H=11.065; df=2; **p=0.004**	7 (5; 9)	H=5.252; df=2; p=0.072
	CT	20 (20; 20)		10 (9; 12)		6 (5; 8)	
	TT	20 (20; 20)		14 (9; 15)		7 (6; 9)	
	T carriers	20 (20; 20)	U=2.376.0; p=0.479	10 (9; 14)	U=1.698.0; **p=0.003**	6 (5; 8)	U=2.001.0; p=0.071
	CC	20 (20; 20)		13 (11; 15)		7 (5; 9)	
	C	20 (20; 20)	U=6.217.0; p=0.310	13 (10; 15)	U=5.061.0; **p=0.014**	7 (5; 9)	U=5.750.0; p=0.185
	T	20 (20; 20)		11 (9; 14)		6 (5; 9)	
Smokers BDNF rs56164415	CC	20 (20; 20)	H=0.306; df=2; p=0.858	13 (10; 15)	H=7.275; df=2; **p=0.025**	7 (6; 10)	H=2.124; df=2; p=0.346
	CT	20 (20; 20)		9 (9; 10)		6 (5; 8)	
	TT	20 (20; 20)		14 (14; 14)		7 (7; 7)	
	T carriers	20 (20; 20)	U=175.5; p=0.771	9 (9; 11)	U=86.0; **p=0.024**	6 (5; 8)	U=123.5; p=0.167
	CC	20 (20; 20)		13 (10; 15)		7 (6; 10)	
	C	20 (20; 20)	U=433.0; p=0.886	12 (10; 15)	U=267.0; p=0.063	7 (5; 10)	U=321.0; p=0.199
	T	20 (20; 20)		10 (9; 13)		7 (5; 8)	

biomolecules

MDPI

Non-smokers BDNF rs56164415	CC	20 (20; 20)	H=0.410; df=2; p=0.815	13 (11; 15)	H=0.989; df=2; p=0.610	7 (5; 9)	H=2.806; df=2; p=0.246
	CT	20 (20; 20)		14 (11; 16)		5 (4; 6)	
	TT	20 (20; 20)		15 (15; 15)		10 (10; 10)	
	T carriers	20 (20; 20)	U=42.0; p=0.522	15 (11; 16)	U=33.5; p=0.390	6 (4; 10)	U=43.0; p=0.766
	CC	20 (20; 20)		13 (11; 15)		7 (5; 9)	
	C	20 (20; 20)		13 (11; 15)		6 (5; 9)	
